# Shaping Ability of Reciprocating and Rotary Systems After Root Canal Retreatment: a CBCT Study

**DOI:** 10.1590/0103-6440202204772

**Published:** 2022-04-29

**Authors:** Eduardo Hideki Suzuki, Emílio Carlos Sponchiado-Júnior, Mariana Travi Pandolfo, Lucas da Fonseca Roberti Garcia, Fredson Márcio Acris de Carvalho, André Augusto Franco Marques

**Affiliations:** 1 School of Dentistry, Federal University of Amazonas, Manaus, AM, Brazil.; 2 Department of Dentistry, Endodontics Division, Health Sciences Center, Federal University of Santa Catarina, Florianópolis, Santa Catarina, Brazil.; 3 Superior School of Health Sciences, State University of Amazonas, Manaus, AM, Brazil.

**Keywords:** Apical transportation, centering ability, rotary systems, reciprocating motion, endodontic retreatment

## Abstract

The purpose of this in vitro study was to evaluate the shaping ability of reciprocating and continuous rotary systems after root canal retreatment. After preparation and root canal filling, mesial canals of 54 mandibular molars were distributed into 3 groups (n=18), according to the filling material removal and re-instrumentation protocols: WOG group - WaveOne Gold system; PTN group - ProTaper Next system; and PTU group - ProTaper Universal system. Cone-beam computed tomographic (CBCT) images acquisition of the mesial root canals was performed at different moments: (1) before instrumentation (unprepared root canals), (2) after preparation and filling, (3) after filling material removal and (4) re-instrumentation. The apical transportation (AT), centering ability (CA) and change in root canal diameter were assessed by CBCT analysis. The remaining filling material quantification was performed by radiographic examination. The statistical analyses were performed using the 3-way ANOVA, Tukey-Kramer, Kruskal-Wallis and Dunn multiple Comparison tests (p<0.05). The tested instruments did not show full CA (=1.0). PTN group had greater AT at the 5th mm in comparison with the WOG group (p<0.05). After re-instrumentation, WOG group had greater root canal diameter change at the 1st and 5th mm than PTN and PTU groups (p<0.05). There was no significant difference among groups when comparing the amount of remaining filling material after re-instrumentation (p>0.05). The tested systems provided minimal alteration in root canal morphology at the apical portion after root canal retreatment. However, WOG promoted greater change in root canal diameter.

## Resumo

O objetivo deste estudo *in vitro* foi avaliar a capacidade de modelagem de sistemas rotatórios e reciprocantes após o retratamento do canal radicular. Após o preparo e obturação do canal radicular, os canais mesiais de 54 molares inferiores foram distribuídos em 3 grupos, de acordo com os protocolos de remoção do material obturador e re-instrumentação: (n=18): grupo WOG - sistema WaveOne Gold; Grupo PTN - sistema ProTaper Next; e grupo PTU - sistema ProTaper Universal. A análise das imagens de tomografia computadorizada de feixe cônico foi realizada em diferentes momentos: (1) antes da instrumentação (canais radiculares não preparados), (2) após o preparo e obturação, (3) após a remoção do material obturador e (4) re-instrumentação. O transporte apical (TA), a capacidade de centralização (CC) e a mudança no diâmetro do canal radicular foram avaliados por análise tomográfica. A quantificação do restante do material obturador foi realizada por exame radiográfico. As análises estatísticas foram realizadas utilizando os testes de ANOVA de 3 fatores, Tukey-Kramer, Kruskal-Wallis e Comparações Múltiplas de Dunn (p<0,05). Os instrumentos não apresentaram CC perfeita (=1,0). PTN apresentou maior TA no 5º mm em comparação ao grupo WOG (p<0,05). Após a re-instrumentação, o grupo WOG apresentou maior aumento no diâmetro do canal radicular no 1° e 5° mm do que os grupos PTN e PTU. Não houve diferença significativa entre os grupos em relação à remoção do material obturador (p>0,05). Os sistemas testados proporcionaram alteração mínima na morfologia do canal radicular na porção apical após o retratamento do canal radicular. No entanto, WOG promoveu maior alteração no diâmetro do canal radicular.

## Introduction

Despite the evolution of the instruments used for endodontic treatment, it is still possible to observe in many cases the perpetuation of periapical lesions and painful symptoms, in which the endodontic retreatment is indicated ^1^ ^,^ ^2^.

The endodontic retreatment consists in removing the filling material from the root canal space by manual or mechanized instruments, followed by its re-instrumentation ^1^ ^,^ ^2^ ^,^ ^3^. This procedure aims to eliminate microorganisms that may have been left or introduced into the root canal system during the first intervention ^1^ ^,^ ^2^ ^,^ ^3^ ^,^ ^4^ ^,^ ^5^.

Mechanized endodontic instruments manufactured from conventional Ni-Ti and thermal treated-Ni-Ti alloys enable safe and faster filling material removal and re-instrumentation of root canals with accentuated curvatures ^6^ ^,^ ^7^ ^,^ ^8^ ^,^ ^9^ ^,^ ^10^ ^,^ ^11^. Among these systems, the ProTaper Next rotary system (Dentsply-Maillefer, Ballaigues, Switzerland) stands out, as well as its predecessor, ProTaper Universal (Dentsply-Maillefer) ^3^ ^,^ ^8^. Although they were developed for root canals preparation, they have been successfully used for filling material removal and re-instrumentation of the root canals ^2^ ^,^ ^3^ ^,^ ^5^ ^,^ ^8^. The ProTaper Next system is manufactured from M-Wire alloy, ensuring to the instruments greater flexibility, decreasing the incidence of cyclic fatigue and torsion fractures when compared to the ProTaper Universal system, manufactured from conventional Ni-Ti alloy ^6^ ^,^ ^7^. 

The WaveOne Gold (Dentsply-Maillefer) reciprocating system was developed to replace the traditional WaveOne (Dentsply-Maillefer) system ^12^. This system has greater flexibility and resistance to cyclic fatigue in comparison with the instruments manufactured from conventional M-Wire alloy, due to a new model of thermal treatment performed in its manufacturing process ^5^ ^,^ ^12^ ^,^ ^13^. 

Azim *et al.* ^5^ assessed the performance of the WaveOne Gold Primary instrument for non-surgical endodontic retreatment. The authors demonstrated that the percentage of remaining filling material was significantly greater for this system in comparison with other instrumentation systems. However, in their study, only the Primary instrument (size 25.07) was used to perform the filling material removal and root canal re-instrumentation. In addition, the authors did not assess the morphological changes promoted by this system in the root canal. In another recent study, Delai *et al.* ^13^ have reported the safety and efficacy of the WaveOne Gold and ProTaper Next systems in removing the filling material. The Primary and Medium (WaveOne Gold) and the X2 and X3 (ProTaper Next) instruments were used for filling material removal and re-instrumentation of the root canals, respectively. However, only the apical transportation promoted by these instrumentation systems during endodontic retreatment was assessed. 

For this reason, the purpose of the present *in vitro* study was to investigate the apical transportation and its direction, the centering ability and the change in the root canal diameter promoted by reciprocating (WaveOne Gold) and continuous rotary systems (ProTaper Universal and ProTaper Next systems) after the filling material removal and re-instrumentation of the root canals. The amount of remaining filling material after endodontic retreatment was also assessed.

The null hypothesis tested was that there would be no difference in the shaping ability of the systems and in the amount of remaining filling material after endodontic retreatment.

## Materials and methods

### Sample size calculation

Based on a pilot study, the sample size was calculated using the G*Power software (version 3.1.9.6) (http://www.psycho.uni-duesseldorf.de/abteilungen/aap/gpower3/) to allow an analysis with α = 0.05, Power (1-ß err prob) = 0.95 and effect size f = 0.80. The ANCOVA (fixed effects, main effects and interactions) statistical test was performed. The type of power analysis was set *a priori* (compute required sample size - given α, power, and effect size). A minimum number of 18 repetitions per experimental group was determined to obtain a reasonable error distribution for statistical analysis.

### Specimens selection

After approval by the Research Ethics Committee of the State University of Amazonas (CAAE No. 82714717.9.0000.5020), 54 freshly extracted mandibular molars were selected. The selection criteria used in this study were: first or second mandibular molar with a root length of 16 mm, two separated and fully formed roots, closed apex and the two mesial canals with independent foramina. Only one of the mesial root canals (mesiobuccal or mesiolingual) was used to perform the study. Teeth with signs of fractures, internal calcification or resorption were discarded from the final sample. 

The teeth were positioned on a digital sensor (New IDA, Dabi Atlante, Ribeirão Preto, SP, Brazil) to perform a radiographic examination in the ortho-radial direction. In order to standardize the radiographic images, the X-ray device (Spectro 70X, Dabi Atlante, Ribeirão Preto, SP, Brazil) was used in accordance with the following parameters: exposure time of 0.4 seconds at a focus-film distance of 10 cm. The radiographic images were analyzed by a single examiner, with the aid of the AutoCAD 2011 software (Autodesk, San Rafael, CA, USA), to measure the angle (from 25° to 70°) and the radius of curvature (from 5 to 10 mm), according to the methods of Schneider ^14^ and Pruett *et al.* ^15^, respectively. Only the roots that have achieved the Schneider's classification ^14^ for severe root canal curvature were included in the final sample.

### Specimens preparation

The teeth were sterilized and stored in plastic receptacles containing distilled water for hydration until use. Next, the teeth were decoronated and the mesial roots were separated with a double-faced diamond disc (KG Sorensen, Barueri, SP, Brazil), coupled to a low-rotation device (Model 605, Kavo, Joinville, SC, Brazil). In order to standardize the biomechanical preparation and to ensure a proper positioning of the specimens during the images acquisition (periapical radiographs and cone-beam computed tomography), the mesial roots were included in self-curing colorless acrylic resin (Dental Vipi Ltda., Pirassununga, SP, Brazil) to form a block from a plaster mold ^4^.

### Cone-beam computed tomographic and digital radiographic images acquisition

Before root canals instrumentation (unprepared root canals), a cone-beam computed tomographic (CBCT) assessment (PaX-i3D, Vatech, Hwaseong-si, Gyeonggi-do, South Korea) of the original trajectories of the root canals at the apical third was performed. The tomographic assessment was also performed after the root canals preparation and filling, after the filling material removal and after the root canals re-instrumentation (four different operatory moments), as described in the study by Delai et al. ^13^. The apical transportation and its direction, centering ability and change in the root canal diameter were calculated based on the images obtained at these different moments. The images acquisition was performed according to the following parameters: source of X-ray with valve voltage of 60-90 kVp, valve current 2-15 mA, focal point of 0.7 mm and 0.4 mm voxel. 

In addition to the tomographic images, digital periapical radiographic images of the root canals were taken in the mesio-distal direction (Spectro 70X, Dabi Atlante) at the same different moments to quantify the amount of remaining filling material after the endodontic retreatment.

### Instrumentation and root canal filling

The root canals were negotiated with a size 10 K-type instrument (Dentsply-Maillefer) until its tip was visualized in the apical foramen. Then, the working length was stablished 1 mm up to the apical foramen. The anatomical diameter of the root canals should be compatible with a size 15 K-type instrument (Dentsply-Maillefer), inserted in the working length. The ProTaper Universal rotary system (Dentsply-Maillefer) was used to prepare the root canals of all specimens, with the following sequence of instruments: Sx (19.04), S1 (18.02), S2 (20.04) and F1 (20.07), at the working length, coupled to a 6:1 contra-angle handpiece (Sirona SN S 12345, VDW GmbH), driven by an electric motor (VDW Gold, VDW GmbH, Munich, Germany) in rotary motion, in pre-determined torque and speed. The instrument Sx was used for pre-flaring of the cervical third; S1 and S2 instruments were used to prepare the middle third; and F1 for apical finishing. The intermittent irrigation of the root canals was performed with 2.5% sodium hypochlorite solution (NaOCl) (Biodinâmica, Ibiporã, PR, Brazil). Afterwards, 3 mL of 17% EDTA solution (Biodinâmica) were used for 3 minutes, followed by final irrigation with 1 mL of 2.5% NaOCl solution. The root canals were dried with absorbent paper points (Dentsply-Maillefer) and filled with the ProTaper F1 gutta-percha master cone (Dentsply-Maillefer) and AH Plus sealer (Dentsply-Maillefer) by the Tagger hybrid technique.

After root canal filling, the teeth were submitted to new CBCT and periapical radiographic analysis. Next, the root canals entrance was sealed with a temporary restorative material, and the teeth were stored for 30 days under 100% humidity at 37°C. After this period, the teeth were randomly distributed into 3 experimental groups (n=18), using the simple casual sample technique, with the aid of the Microsoft Excel 2010 software (Microsoft Corporation, Redmond, WA, USA).

### Root canal filling removal and re-instrumentation

The specimens were distributed according to the system used for filling material removal and root canal re-instrumentation, as follows:

 WOG Group: filling material removal with the Primary instrument (25.07) and re-instrumentation of the root canal with the Medium instrument (35.06), both of the WaveOne Gold reciprocating system.

 PTN Group: filling material removal with the X2 instrument (25.06) and re-instrumentation of the root canal with the X3 instrument (30.07) of the ProTaper Next system.

 PTU Group: filling material removal with the F2 instrument (25.08) and re-instrumentation of the root canal with the F3 instrument (30.09) of the ProTaper Universal system.

Initially, Largo burs (Dentsply-Maillefer) (sizes 1 and 2) were used to perforate the gutta-percha at the cervical third of the root canal to facilitate the action of the subsequent instruments ^2^. Next, the instruments were gradually inserted into the root canal in the apical direction (3-mm amplitude), with gently in-and-out movements, under moderate pressure, until reach the working length. Each instrument was used to prepare a maximum of three specimens from the same experimental group, being cleaned with sterile gauze after each advance into the root canal. The root canals were irrigated with 1 mL of 2.5% NaOCl solution, with a disposable plastic syringe (Ultradent Products Inc., South Jordan, UT, USA) and a yellow NaviTip needle (Ultradent Products Inc.), after each gradual advance of the instrument. At the end of the root canal preparation, the excess of 2.5% NaOCl solution was aspirated (CapillaryTip, Utradent Products Inc.). Next, the root canals were irrigated with 3 mL of 17% EDTA for 3 minutes, followed by 2 mL of distilled water, and dried with absorbent paper points (Dentsply-Maillefer).

After filling material removal and after re-instrumentation of the root canals, the specimens were submitted to the last two tomographic and radiographic examinations, as described above.

### Apical transportation analysis and transport direction

The apical transportation was measured at the 1^st^, 3^rd^ and 5^th^ millimeters of the apical third, generating axial images of 1 mm-thick for each root canal. The images were analyzed with the aid of the OsiriX MD software (OsiriX Imaging Software, http://www.osirix-viewer.com). For images standardization, the following protocol was used to minimize the influence of the filling material on the artifact’s creation: the “3D View visualization” tool was selected, followed by the “3D MPR” option; afterwards, the contrast and the “Default WL&WW” intensity were applied. To assign color to the images, the CLUT tool was used in the “Perfusion” mode and the opacity in the “Smooth Table” option.

**Figure 1:**
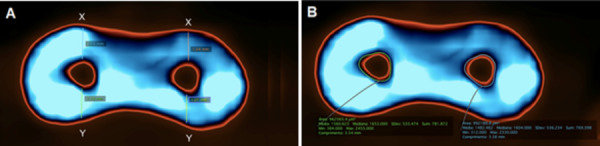
Representative tomographic images of the root canals visualized in the Osirix MD software. (a) Measurement of apical transportation performed before root canal preparation to be applied in the formula: AT = (X1 - X2) - (Y1 - Y2). (b) Root canal area outlined to determine its limit.

The diameter of the root canal walls after the initial instrumentation, after the filling material removal and re-instrumentation was calculated. X1 is the measurement of the external mesial wall (non-instrumented canal); X2 is the measurement of the external mesial wall (instrumented canal); Y1 is the measurement of the external distal wall (non-instrumented canal); and Y2, the measurement of the external distal wall (instrumented canal). The mesial and distal measurements of the topograms were performed from the outermost portion of the root to the outer portion of the root canal, considering as a reference point the most central portion of the root canal in the mesio-distal direction (Figure 1a).

To calculate the apical transportation (AT), the formula proposed by Gambill *et al.* ^16^ was used: AT = (X1 - X2) - (Y1 - Y2). When the AT value was equal to zero, there was no transportation; when the AT value was negative, transportation occurred in the distal direction, and when AT was positive, transportation occurred in the mesial direction.

### Centering ability

The centering ability (CA) of the instruments was calculated at the 1^st^, 3^rd^ and 5^th^ millimeters of the apical third, based on the values obtained during the apical transportation measurement. The values were applied to the formula described by Gambill et al. ^21^: CA = X1 - X2 / Y1 - Y2 or CA = Y1 - Y2 / X1 - X2. Values close to 1 indicated acceptable centering ability of the instrument, and values close to 0, less ability of the instrument to remain centralized within the root canal.

### Change in root canal diameter

With the aid of the OsiriX MD software (OsiriX Imaging Software), the root canal diameter was calculated at the 1^st^, 3^rd^ and 5^th^ millimeters of the apical third. Initially, the root canal area was outlined to determine its limit (Figure 1b). The change in root canal diameter was calculated by subtracting the diameter of the canals after the initial instrumentation, after the filling material removal and after re-instrumentation from the area before to preparation. The values obtained in millimeters after subtraction were transformed into percentage values.

### Remaining filling material measurement

The digital radiographic images (New IDA, Dabi Atlante) of the root canals were used to measure the amount of remaining filling material. An integration grid (0.8 mm x 0.8 mm) was superimposed to the radiographic images after root canal filling, after filling material removal and after re-instrumentation of the root canals. With the integration grid superimposed, the number of quadrants containing remaining filling material (RFM) was quantified, using the following equations: RFM = {(B x 100)/A} to measure the percentage of remaining filling material after the removal procedure; and RFM = {(C x 100)/A} to measure the percentage of remaining filling material after the re-instrumentation of the root canals.

### Remaining filling material measurement

The digital radiographic images (New IDA, Dabi Atlante) of the root canals were used to measure the amount of remaining filling material. An integration grid (0.8 mm x 0.8 mm) was superimposed to the radiographic images after root canal filling, after filling material removal and after re-instrumentation of the root canals. With the integration grid superimposed, the number of quadrants containing remaining filling material (RFM) was quantified, using the following equations: RFM = {(B x 100)/A} to measure the percentage of remaining filling material after the removal procedure; and RFM = {(C x 100)/A} to measure the percentage of remaining filling material after the re-instrumentation of the root canals.

All analyses were performed by a blinded and previously calibrated dental radiologist, with more than 5 years of experience in dentomaxillofacial radiographic and tomographic imaging. The examiner had all the time necessary to evaluate the CBCT scans and the radiographs. In order to minimize bias due to interpretation difficulties and to verify whether the examiner was consistent in his/her opinions, 25% of the sample was randomly selected, assessed and reassessed at two different periods, with an interval of 15 days between the first and the second assessments. The data obtained at this stage were submitted to statistical testing to ensure their validity and reproducibility. The intraclass correlation coefficient (ICC) test was used to assess the intra-examiner agreement until the establishment of ICC indexes above 0.75, considered excellent.

### Statistical analysis

The data for apical transportation, centering ability, change in root canal diameter and remaining filling material measurement were submitted to statistical analysis with the aid of the GraphPad InStat software (GraphPad Software, La Jolla, CA, USA). The normality of the sample was confirmed by the Kolmogorov-Smirnov test (p>0.05). The 3-way ANOVA test for independent factors (apical millimeter, root canal - mesiolingual or mesiobuccal - and instrumentation system) was initially applied to the data and complemented by the *post hoc* Tukey-Kramer test (p<0.05). For apical transportation, the data did not present a normal distribution. The Kruskal-Wallis and the Dunn's Multiple Comparisons tests were applied to the data (p<0.05).

## Results

### Apical transportation

The mean values (mm) for apical transportation are in Table 1. 

Significant difference (p<0.05) was found only at the 5^th^ millimeter for WOG and PTN systems, during the filling material removal. The PTN system had greater transportation than WOG group.

**Table 1:** Mean values (mm) for apical transportation

System/apical millimeter		Filling material removal	Root canal re-instrumentation
WOG	1^st^	0.011	0.219
	3^rd^	-0.211	-0.055
	5^th^	0.135*	-0.002
PTN	1^st^	0.068	-0.016
	3^rd^	-0.098	-0.208
	5^th^	-0.128*	0.144
PTU	1^st^	0.158	-0.171
	3^rd^	-0.006	-0.096
	5^th^	-0.044	0.113

(*) indicates statistically significant difference among groups (level of significance = 5%). Kruskal-Wallis and the Dunn's Multiple Comparisons tests, p<0.05.

### Apical transportation direction

The values for the apical transportation direction are in Table 2.

The highest percentage of apical transportation was towards the mesial direction (51.23%), when compared to the distal direction (47.68%). A total of 7 points (1.08%), among the 648 evaluated, did not present transportation to mesial or distal directions.

**Table 2:** Apical transportation direction after the filling material removal and root canal re-instrumentation

Apical transportation direction after filling material removal									
	Mesiolingual			Mesiobuccal			Total		
System/apical millimeter	Mesial	Distal	Absence	Mesial	Distal	Absence	Mesial	Distal	Absence
WOG - 1^st^ mm	10	8	0	10	8	0	20	16	0
WOG - 3^rd^ mm	7	11	0	5	13	0	12	24	0
WOG - 5^th^ mm	10	7	1	13	5	0	23	12	1
PTN - 1^st^ mm	14	4	0	7	10	1	21	14	1
PTN - 3^rd^ mm	10	8	0	9	9	0	19	17	0
PTN - 5^th^ mm	10	8	0	7	11	0	17	19	0
PTU - 1^st^ mm	13	5	0	13	5	0	26	10	0
PTU - 3^rd^ mm	7	11	0	9	9	0	16	20	0
PTU - 5^th^ mm	6	11	1	9	9	0	15	20	1
Apical transportation direction after root canal re-instrumentation									
WOG - 1^st^ mm	11	7	0	12	6	0	23	13	0
WOG - 3^rd^ mm	8	10	0	11	7	0	19	17	0
WOG - 5^th^ mm	10	8	0	7	11	0	17	19	0
PTN - 1^st^ mm	10	6	2	7	11	0	17	17	2
PTN - 3^rd^ mm	6	12	0	9	9	0	15	21	0
PTN - 5^th^ mm	11	7	0	11	7	0	22	14	0
PTU - 1^st^ mm	8	10	0	5	13	0	13	23	0
PTU - 3^rd^ mm	7	11	0	10	7	1	17	18	1
PTU - 5^th^ mm	10	8	0	10	7	1	20	15	1
						Total	332	309	7
							(51.23%)	(47.68%)	(1.08%)

The three tested systems had a greater tendency towards transport to the mesial, after filling material removal. After re-instrumentation of the root canals, the WOG and PTN systems had greater tendency towards transport to the mesial and PTU system towards distal.

### Centering ability

None of the tested systems presented adequate centering ability (CA=1.0), with no statistically significant difference among them (p>0.05). The PTN system had centering ability closer to 1 during the filling material removal. The mean values for centering ability may be seen in Table 3.

**Table 3:** Mean values (mm) for centering ability

System	Filling material removal	Root canal re-instrumentation
WOG	0.14	-0.755
PTN	0.833	-0.271
PTU	-0.26	0.003

There was no statistically significant difference among groups (level of significance = 5%). 3-way ANOVA and Tukey-Kramer tests, p<0.05.

### Change in root canal diameter

The mean values for the change in root canals diameter are in Table 4.

**Table 4:** Mean values (%) for change in root canal diameter

	After filling material removal			Removal x re-instrumentation			After re-instrumentation		
System	1^st^ mm	3^rd^ mm	5^th^ mm	1^st^ mm	3^rd^ mm	5^th^ mm	1^st^ mm	3^rd^ mm	5^th^ mm
WOG	27.40ª	25.58ª	27.37ª	37.18ª	32.86ª	40.55ª	73.79ª	67.18ª	72.71ª
PTN	24.92^b^	26.31ª	25.70ª	26.89^b^	27.56^b^	26.06^b^	59.28^b^	60.13ª	57.58ª
PTU	25.70^b^	25.14ª	26.94ª	27.90^b^	27.79^b^	24.88^b^	60.56^b^	60.05ª	58.37ª

Different lowercase letters in columns means statistically significant difference among groups (level of significance = 5%). 3-way ANOVA and Tukey-Kramer tests, p<0.05.

The WOG system promoted the highest increase in root canal diameter in comparison with the PTN and PTU systems, after the filling material removal and re-instrumentation of the root canals, both at the 1^st^ apical millimeter (p<0.05). There was no statistically significant difference between PTN and PTU groups (p>0.05). After re-instrumentation of the root canal, a significant difference was observed between the 1^st^ and 5^th^ millimeters (p<0.05).

When comparing the change in root canal diameter after filling material removal and the re-instrumentation, it was possible to observe statistically significant difference (p<0.001) at the 1^st^, 3^rd^ and 5^th^ millimeters for the WOG group, when compared to the PTN and PTU groups (Table 4).

### Remaining filling material

**Figure 2:**
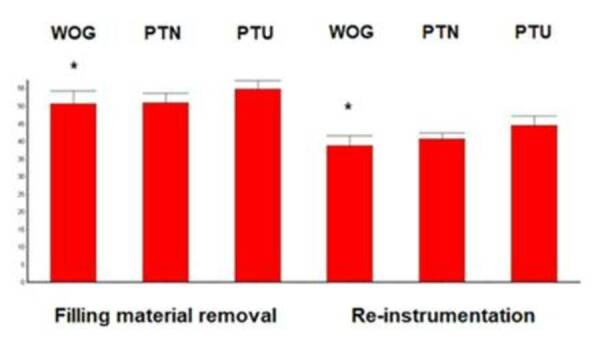
Graphic representation of the remaining filling material after removal and after re-instrumentation of the root canals. (*) over bars indicate statistically significant difference (3-way ANOVA Tukey-Kramer tests, p<0.05).

There was no significant difference among groups when comparing the remaining filling material after removal and after the re-instrumentation of the root canals (p>0.05). There was statistically significant difference (p<0.05) only in the intra-group comparison for WOG, in which the instrument used for re-instrumentation (Medium) promoted greater filling material removal than the Primary instrument (Figure 2).

## Discussion

The advent of single-file systems provided faster endodontic treatment protocols with the use of a reduced number of instruments ^17^. This may also be applied to non-surgical endodontic retreatment procedures, in which instruments of different motions, designs, tapers and NiTi alloys have been replaced the traditional manual instruments in this treatment modality ^8^ ^,^ ^13^ ^,^ ^18^. 

The purpose of this *in vitro* study was to evaluate the shaping ability of different instrumentation systems after the filling material removal and root canal re-instrumentation. The amount of remaining filling material after endodontic retreatment was also assessed.

According to the results obtained, the null hypothesis tested was partially accepted, as the different systems presented similar performance with regard to the apical transportation, centering ability and their ability in removing the filling material after endodontic retreatment. However, the WaveOne Gold system promoted greater change in root canal diameter than the ProTaper Universal and ProTaper Next systems. 

Several studies have reported that no instrumentation system (manual or mechanized) or technique are able to completely remove the filling material from the root canal system during the endodontic retreatment ^2^ ^,^ ^3^ ^,^ ^5^ ^,^ ^8^ ^,^ ^13^. For this reason, we decided to use an instrument of greater diameter to perform the re-instrumentation of the root canals, in order to achieve greater filling material removal ^13^. The protocols for endodontic retreatment were based on the study by Delai *et al.* ^13^, in which two instruments from each system were used: the first instrument had a smaller tip diameter (X2 - 25.06 - ProTaper Next; Primary - 25.07 - WaveOne Gold; and F2 - 25.08 ProTaper Universal), and it was used for filling material removal. The second instrument from the same system had a larger tip diameter (X3 - 30.07 - ProTaper Next; Medium - 35.06 WaveOne Gold; and F3 - 30.09 - ProTaper Universal), and it was used for root canal re-instrumentation (shaping). Therefore, in addition to the filling removal capacity of these protocols, the shaping ability of these systems and their effect on the root canal morphology needed to be tested, since another instrument, with a greater tip diameter, was used for root canal shaping.

Conversely to the study by Delai *et al.* ^13^, which assessed only the apical transportation during the endodontic retreatment, in the present study, we evaluated the transportation and its direction, the centering ability and the change in the root canal diameter promoted by the WaveOne Gold, ProTaper Universal and ProTaper Next systems after the filling material removal and re-instrumentation of the root canals. To the best of our knowledge, no studies so far have assessed all factors involving the shaping ability of these systems, especially during endodontic retreatment.

The retreatment protocol of the present study was performed on mesial roots of mandibular molars with two canals and independent foramina, however, only one mesial canal (mesiobuccal or mesiolingual) from each root was used to carry out the investigation. In addition, only roots with severe curvature (ranging from 25° to 70°) ^14^ were included in the final sample, following the criteria adopted in previous studies ^8^ ^,^ ^13^. This sample selection aimed to eliminate any curvature variation that might compromise the analysis and to increase the risk of bias ^8^ ^,^ ^13^. 

Among the several methods used to assess the shaping ability of different instrumentation systems ^8^ ^,^ ^11^ ^,^ ^13^ ^,^ ^19^, CBCT has been widely used because it is a method capable of obtaining undistorted three-dimensional images of an individual tooth or teeth, before and after the experimental procedures, in a non-destructive way, preserving the structure of the specimen ^6^ ^,^ ^20^ ^,^ ^21^. According to Borges *et al.* ^22^, CBCT may be as accurate as micro-computed tomography in the assessment of morphological characteristics of extracted human teeth, proving to be a reliable and non-invasive measurement tool to explore in detail the root canal system morphology ^6^ ^,^ ^18^ ^,^ ^20^. In another study, Bunn *et al.* ^23^ have reported that CBCT overestimates the radicular dentin thickness by 0.20 mm. However, this measurement discrepancy may be considered clinically acceptable and endorses the reliable performance of this type of tomographic diagnostic tool.

However, CBCT also has limitations ^23^ ^,^ ^24^. Gutta-percha and sealer generate a low-beam hardening artifact, hindering a proper measurement of the amount of remaining filling material after root canal re-instrumentation ^24^. According to Vizzoto *et al.* ^24^, the influence of the filling material on the presence of artifacts is highly dependent on the CBCT voxel size used during the images acquisition. In the present research, a pilot study testing the accuracy of different voxel-size images was previously performed, and the dental radiologist (examiner) confirmed the presence of artifacts, compromising the reliability of the analysis. For this reason, digital radiographic images were used to quantify the remaining filling material. The authors opted for this method to avoid any interference, which might hinder the analysis of the results ^24^.

The mechanical preparation of the root canal walls occurs most frequently in three different areas and the first of these areas is the apical third, in which the final portion of the instrument enlarges the external wall of the canal ^21^. It was observed in the present study a greater tendency towards transport to the mesial direction, that is, to the external portion of the root, at the anti-furcation region. This finding is in agreement with several other studies ^11^ ^,^ ^19^ ^,^ ^21^. However, according to You *et al.* ^25^, the transportation may abruptly change its direction according to the apical position assessed. The apical transportation in different directions in the same root canal is directly associated with the angle and the radius of curvature of the root ^25^. Greater apical transportation towards the mesial wall occurs as the distal wall acts in the anti-furcation direction and forces the instrument against the mesial wall of the root canal ^11^ ^,^ ^19^ ^,^ ^21^ ^,^ ^25^. 

When evaluating the apical transportation after the filling material removal and the re-instrumentation of the root canals, no statistically significant difference was observed at the 1^st^ and 3^rd^ apical millimeters among the tested systems. However, there was statistical difference at the 5^th^ mm of the mesiobuccal canal after the filling material removal when the WOG and PTN groups were compared. This result may be explained by the greater flexibility of the WOG system and the controlled memory of the alloy from which the instrument was fabricated ^5^ ^,^ ^13^. These characteristics of the WOG system provide a more centralized instrumentation, with less tension in the anti-furcation direction in roots with more accentuated curvatures, such as those used in present study ^5^ ^,^ ^13^. 

It is also valid to state that the maximum stress supported by any type of NiTi alloy is directly affected by the cross-section configuration of the instrument ^20^ ^,^ ^21^ ^,^ ^25^. Thus, the manufacturers of the WOG system designed the instrument with a unique parallelogram configuration, with only one or two cutting edges, depending on the location, with the objective of following the root canal curvature with greater precision, preventing apical transportation.

Furthermore, most of the samples assessed in this study had apical transportation values after filling material removal and root canal re-instrumentation lower than 0.06 mm, regardless the instrumentation system used, which is considered clinically irrelevant ^21^.

It is consensus in the scientific literature that values closer to 0 (zero) mean lesser ability of the instrument to remain centralized inside the root canal, and the closer to 1 (one), the greater this ability would be ^16^. It is important to point out that the presence of filling material occupying the root canal lumen, as observed in most samples in the present study, may be considered as an interference in the instrument's path towards the apical foramen ^6^. Furthermore, although no statistically significant difference was found among the tested systems, the PTN system had a centering ability closer to 1 after the filling material removal. This result may be related to the eccentric movement performed by the PTN instruments during preparation, because of their decentralized nucleus ^7^ ^,^ ^10^. Only two cutting-edges of the PTN instruments touch the root canal walls simultaneously, reducing the chances of deviation from the original canal trajectory ^7^ ^,^ ^10^. 

Conversely, after the re-instrumentation, none of the tested systems presented centering ability values close to 1. The instruments used in this operative stage had a larger diameter than the instruments used for filling material removal, increasing the chances of decentralization ^7^ ^,^ ^8^ ^,^ ^13^. 

The WOG system provided significant increase in root canal diameter after re-instrumentation, in comparison with the PTN and PTU systems, at the 1^st^ and 5^th^ apical millimeters. Statistically significant difference was also found when comparing the root canal diameter after filling material removal and re-instrumentation. The WOG system had the highest values at the 1^st^, 3^rd^ and 5^th^ apical millimeters. 

Previous studies have assessed the change in root canal diameter after preparation with instruments with the same tip diameter ^6^ ^,^ ^8^. On the other hand, in the present study, the instrument of the WOG system used for re-instrumentation had a larger tip diameter (350 µm) ^5^ ^,^ ^13^ when compared to the PTN and PTU systems (300 µm) ^8^ ^,^ ^10^ ^,^ ^13^, which might explain the greater increase in the root canal diameter promoted by this system. 

In addition, it was noted a significant increase in the root canal diameter after re-instrumentation with the WOG system in comparison with the root canal diameter after filling material removal. This phenomenon did not occur in PTN and PTU groups and might be explained by the diameter of the instrument of the WOG system used in the re-instrumentation (35.06 - 350 µm), which is 100 µm larger than the instrument used for filling material removal (25.07 - 250 µm) ^13^. Conversely, the diameter of the instruments of the PTN and PTU systems used for re-instrumentation have a diameter only 50 µm larger than the instruments used for filling material removal (250 µm) ^1^ ^,^ ^2^ ^,^ ^7^.

The same was observed in relation to the amount of filling material removal. Despite no system was capable of completely remove the filling material from the root canal, the instrument of the WOG system used for re-instrumentation promoted greater filling material removal than the instrument specifically used for this purpose. This fact might also be explained by the lower dentin/filling material removal promoted by the instrument of smaller diameter (Primary) than the instrument used for re-instrumentation (Medium) ^4^ ^,^ ^21^. In addition, the parallelogram-shaped cross-section of the WOG instruments provides an efficient space for improved cutting, facilitating the transportation of filling material remnants in the coronal direction ^10^.

In spite of the limitations, and according to the experimental conditions and the methods applied in this *in vitro* study, the WaveOne Gold system promoted greater change in root canal diameter than the ProTaper Universal and ProTaper Next systems. However, the tested systems, despite the different apical diameters of the instruments used for root canal re-instrumentation, provided minimal alteration in root canal morphology, maintaining the original shape at the apical portion after endodontic retreatment. In addition, there was no difference among the systems regarding their ability in removing the filling material.
